# Supplementary material for: BMAL1 modulates glutamine supply to control haematopoietic stem and progenitor cell expansion
Source: Development. 2026 Apr 15;153(8):dev204726. doi: 10.1242/dev.204726 (PMC13120679; doi:10.1242/dev.204726)
Supplement: Supplementary information [file develop-153-204726-s1.pdf]

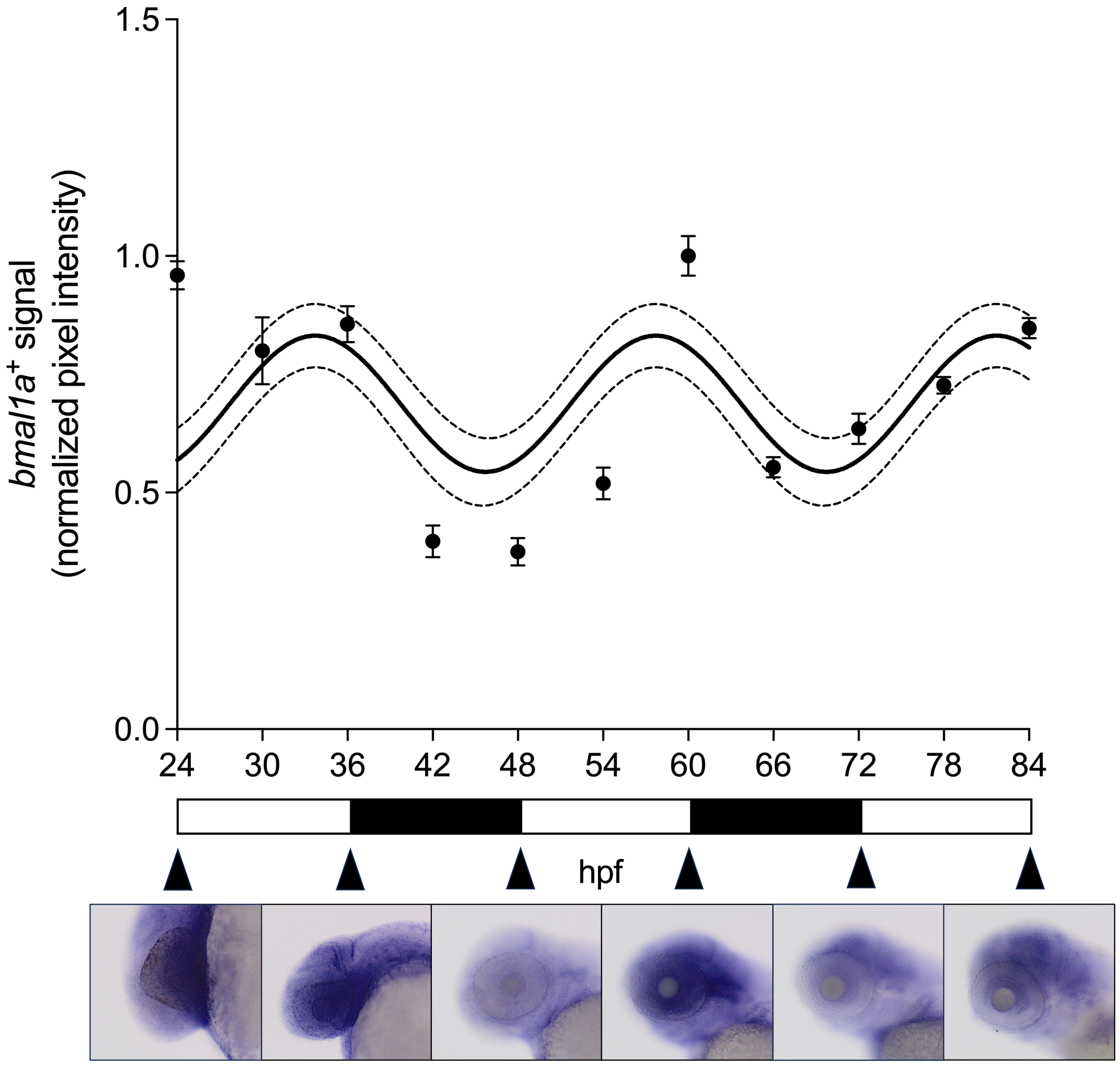

Fig. S1. *bmal1a* is expressed rhythmically in the heads of zebrafish embryos raised in light-dark cycles. Quantification of normalised *bmal1a in situ* hybridisation signal between 24-84 hpf in the heads of wild-type zebrafish raised in light-dark cycles; *n*=9-10 embryos or larvae for each timepoint. Images under the graph are representative *in situ* images of embryo / larvae heads every 12 hours. Circadian rhythmicity was determined by cosinor analysis.

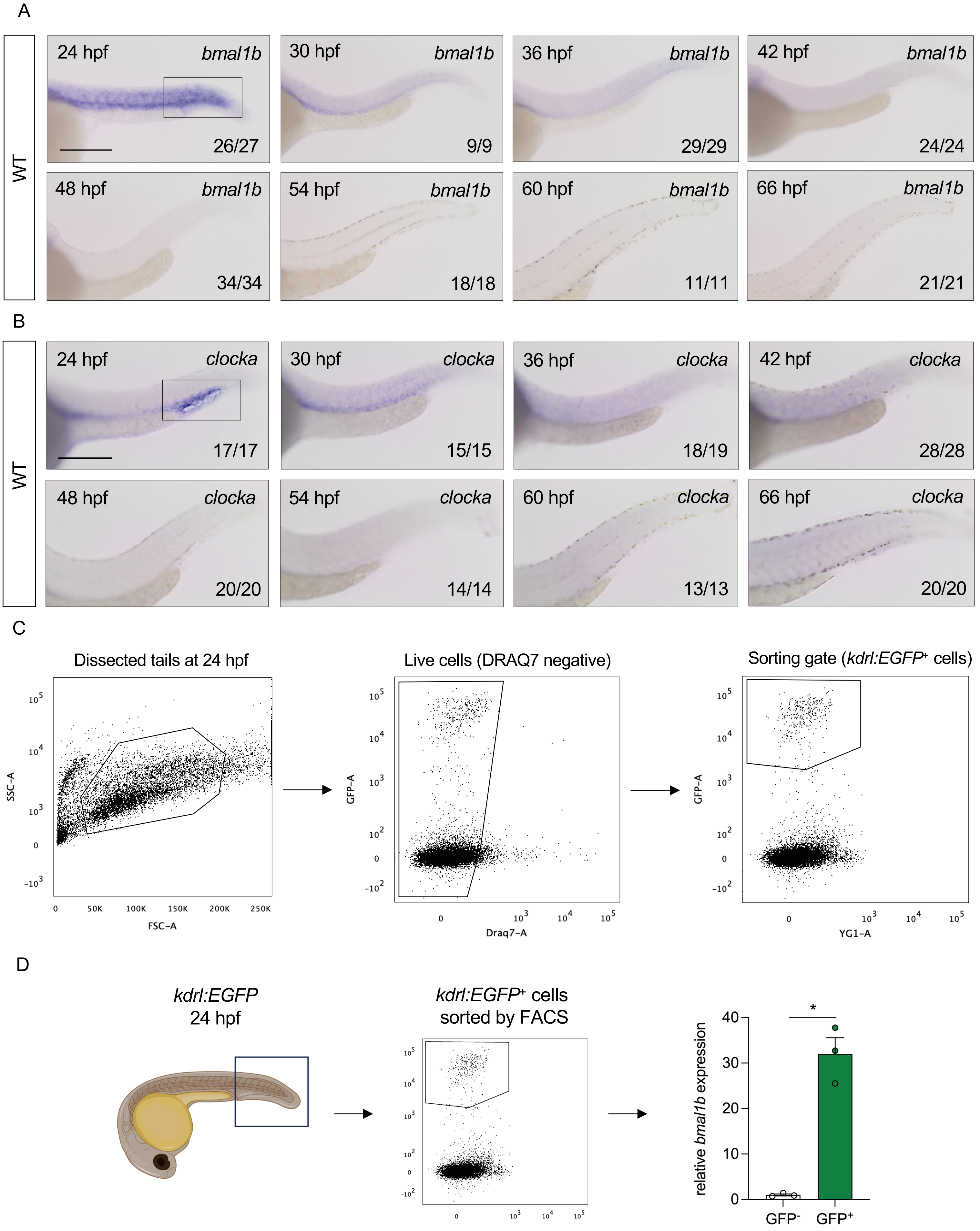

Development • Supplementary information

Fig. S2. Circadian clock genes are expressed in the CHT at 24 hpf. A. *in situ* hybridisation of *bmal1b* from 24-66 hpf in the tails of zebrafish embryos raised in light-dark cycles. **B.** *in situ* hybridisation of *clocka* from 24-66 hpf in the tails of zebrafish embryos raised in light-dark cycles. **C.** Fluorescence activated cell sorting (FACS) plots to depict the strategy used to sort endothelial cells from the tails of *kdrl:EGFP* embryos at 24 hpf. **D.** *kdrl:EGFP* tails were dissected at 24 hpf before endothelial cells were sorted by FACS and *bmal1b* expression was quantified by qPCR; *n*=~50 embryos in triplicate. Rectangles in **A** and **B** indicate the CHT area. Statistical significance between two groups was calculated using unpaired two-tailed Student's *t*-tests assuming equal variance. Scale bars: 200 μm. Created in BioRender by Petzold, T. (2026) https://BioRender.com/y7gmtpg. This figure was sublicensed under CC-BY 4.0 terms.

Development • Supplementary information

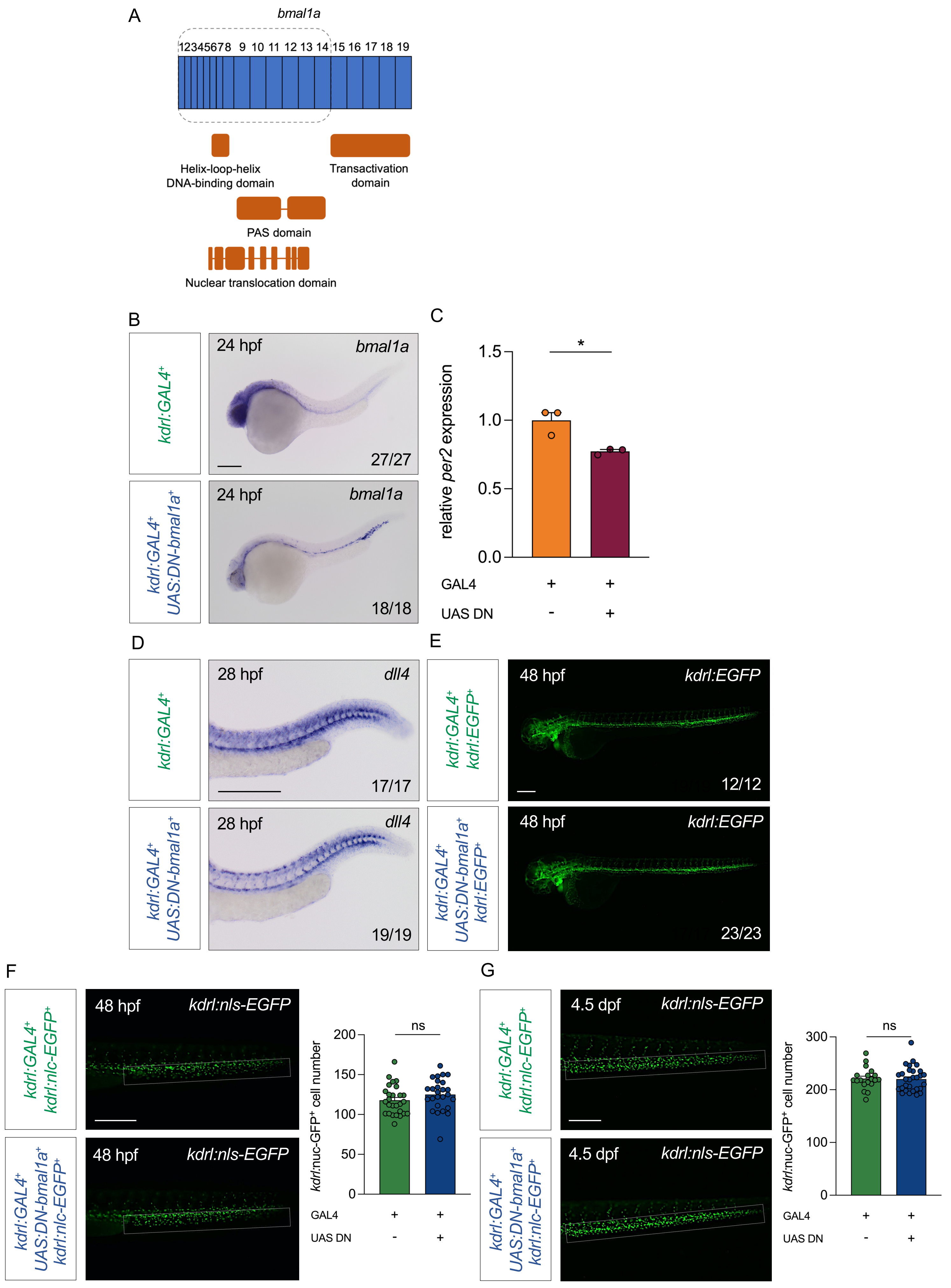

Development • Supplementary information

Fig. S3. Loss of the Bmal1a transactivation domain in endothelial-specific dominant-negative Bmal1a zebrafish embryos reduces target gene expression but does not alter vascular development. A. Schematic showing the region of the *bmal1a* gene (within the dotted line) that was cloned from cDNA for the generation of the *UAS:DN-bmal1a* zebrafish line. Exons are indicated in blue (numbered 1-19) and protein domains are indicated in orange. Note the exclusion of the transactivation domain in the cloned fragment. **B.** *in situ* hybridisation of *bmal1a* in *kdrl:GAL4;UAS:DN-bmal1a* embryos and controls at 24 hpf. **C.** qPCR analysis of *per2* expression in whole *kdrl:GAL4;UAS:DN-bmal1a* embryos and controls at 36 hpf; *n*=~30 embryos in triplicate for each genotype. **D.** *in situ* hybridisation of *dll4* in *kdrl:GAL4;UAS:DN-bmal1a* embryos and controls at 28 hpf. **E.** *kdrl:EGFP* fluorescence in *kdrl:GAL4;UAS:DN-bmal1a;kdrl:EGFP* embryos and controls at 48 hpf. **F.** *kdrl:nls-EGFP* fluorescence and GFP^+ cell quantification in the CHT of *kdrl:GAL4;UAS:DN-bmal1a;kdrl:nls-EGFP* embryos and controls at 48 hpf. **G.** *kdrl:nls-EGFP* fluorescence and GFP^+ cell quantification in the CHT of *kdrl:GAL4;UAS:DN-bmal1a;kdrl:nls-EGFP* larvae and controls at 4.5 dpf. Rectangles in **F** and **G** indicate the CHT area in which cells were quantified. Statistical significance between two groups was calculated using unpaired two-tailed Student's *t*-tests assuming equal variance. Scale bars: 200 µm.

Development • Supplementary information

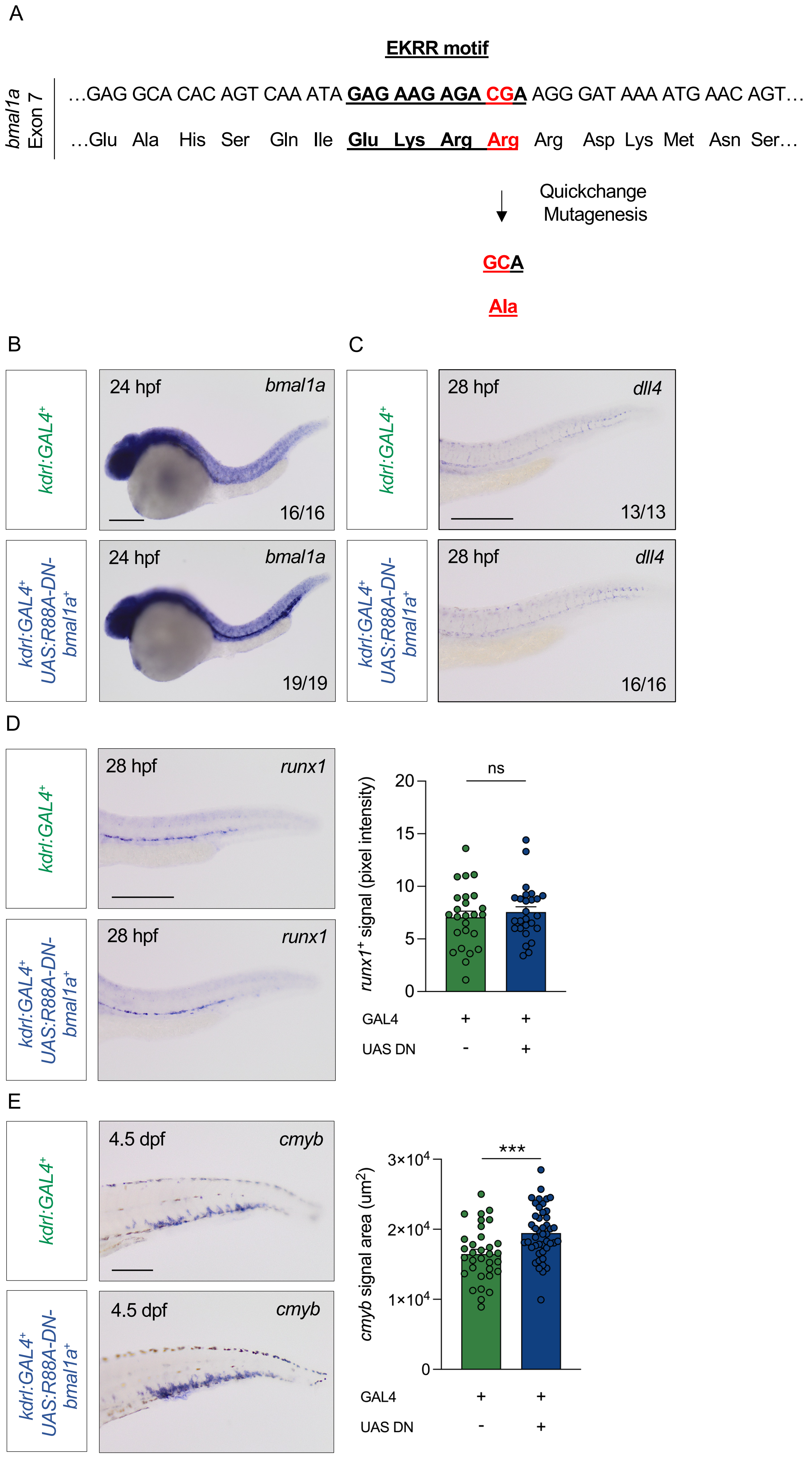

Development • Supplementary information

Fig. S4. Endothelial-specific R88A-*DN-bmal1a* results in an increase in the number of HSPCs in the CHT at 4.5 dpf. A. Experimental schematic showing the R88A amino acid change in the *bmal1a* EKRR motif induced by quickchange mutagenesis. **B.** *in situ* hybridisation of *bmal1a* in *kdrl:GAL4;UAS:R88A-DN-bmal1a* zebrafish embryos and controls at 24 hpf. **C.** *in situ* hybridisation of *dll4* in *kdrl:GAL4;UAS:R88A-DN-bmal1a* embryos and controls at 28 hpf. **D.** *runx1 in situ* hybridisation and quantification in *kdrl:GAL4;UAS:R88A-DN-bmal1a* embryos and controls at 28 hpf. **E.** *cmyb in situ* hybridisation and quantification in *kdrl:GAL4;UAS:R88A-DN-bmal1a* larvae and controls at 4.5 dpf. Statistical significance between two groups was calculated using unpaired two-tailed Student's *t*-tests assuming equal variance. Scale bars: 200 µm.

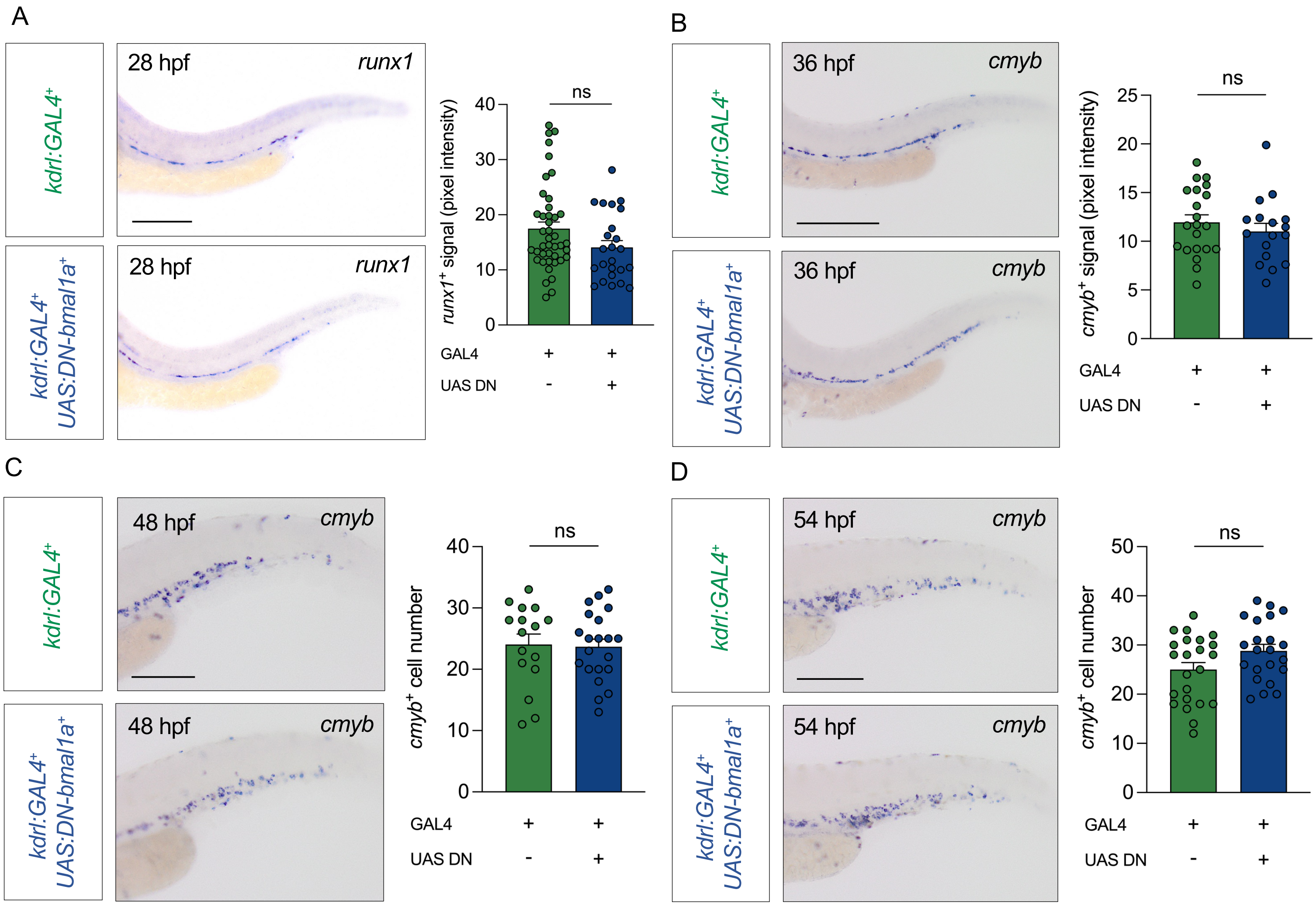

Fig. S5. Endothelial-specific dominant-negative *bmal1a* does not result in an increase in HSPC numbers in the CHT between 28 and 54 hpf. A. *runx1 in situ* hybridisation and quantification in *kdrl:GAL4;UAS:DN-bmal1a* zebrafish embryos and controls at 28 hpf. **B.** *cmyb in situ* hybridisation and quantification in *kdrl:GAL4;UAS:DN-bmal1a* embryos and controls at 36 hpf. **C.** *cmyb in situ* hybridisation and quantification in *kdrl:GAL4;UAS:DN-bmal1a* embryos and controls at 48 hpf. **D.** *cmyb in situ* hybridisation and quantification in *kdrl:GAL4;UAS:DN-bmal1a* embryos and controls at 54 hpf.

Development • Supplementary information

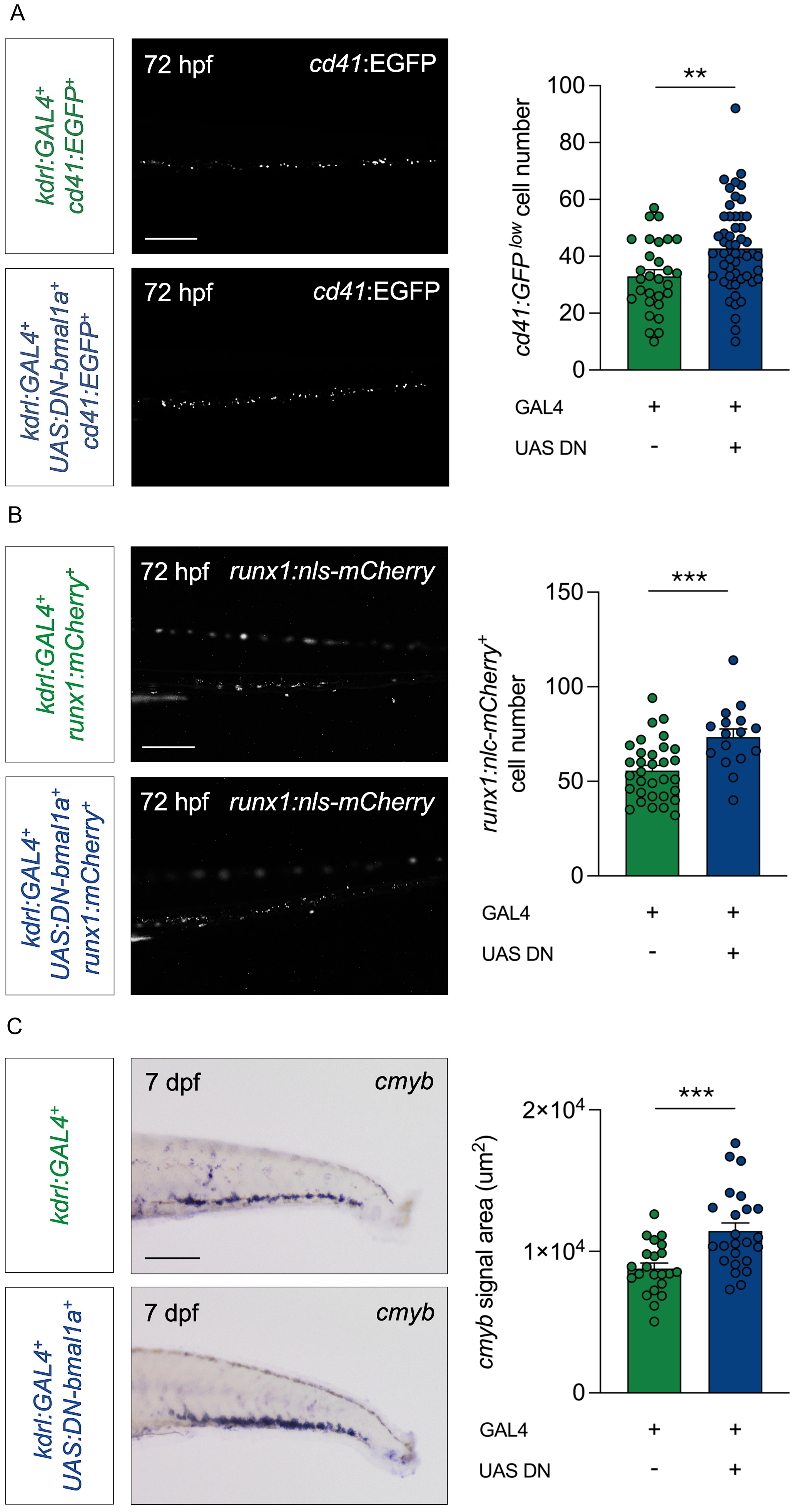

Development • Supplementary information

Fig. S6. Endothelial-specific DN-*bmal1a* larvae have an increased number of HSPCs in the CHT. A. *cd41:EGFP*+ cells and quantification of *cd41:EGFP*low cells in the CHT of *kdrl:GAL4;UAS:DN-bmal1a;cd41:EGFP* zebrafish larvae and controls at 72 hpf. **B.** *runx1:nls-mCherry*+ cells and quantification in the CHT of *kdrl:GAL4;UAS:DN-bmal1a;runx1:nls-mCherry* larvae and controls at 72 hpf. **C.** *cmyb in situ* hybridisation and quantification in *kdrl:GAL4;UAS:DN-bmal1a;cmyb:GFP* larvae and controls at 7 dpf. Statistical significance between two groups was calculated using unpaired two-tailed Student's *t*-tests assuming equal variance. Scale bars: 200 μm.

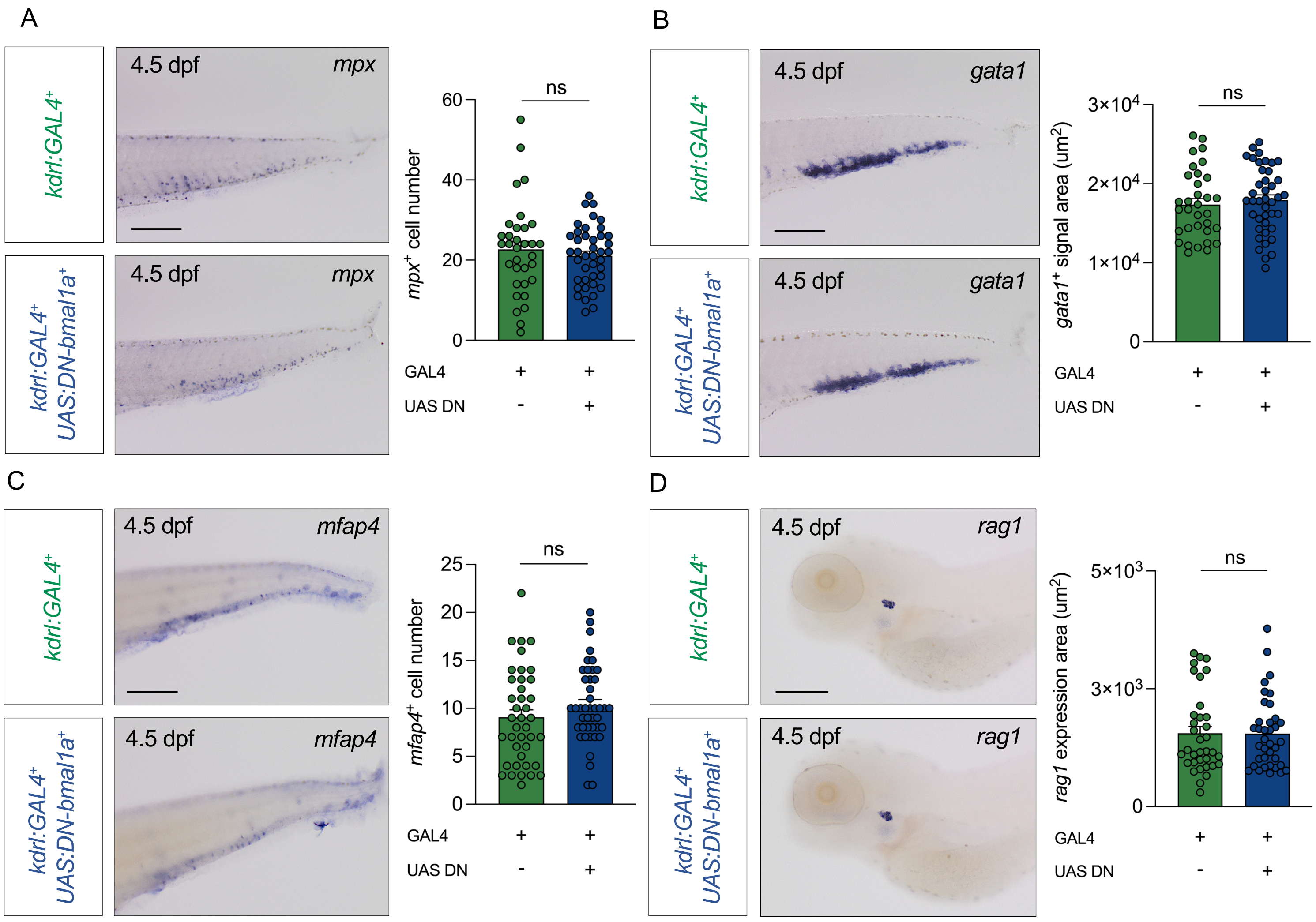

Fig. S7. Endothelial-specific *DN-bmal1a* does not alter neutrophil, erythrocyte, macrophage or T-cell numbers at 4.5 dpf. A. *mpx in situ* hybridisation and quantification in *kdrl:GAL4;UAS:DN-bmal1a* zebrafish larvae and controls at 4.5 dpf. **B.** *gata1 in situ* hybridisation and quantification in *kdrl:GAL4;UAS:DN-bmal1a* larvae and controls at 4.5 dpf. **C.** *mfap4 in situ* hybridisation and quantification in *kdrl:GAL4;UAS:DN-bmal1a* larvae and controls at 4.5 dpf. **D.** *rag1 in situ* hybridisation and quantification in *kdrl:GAL4;UAS:DN-bmal1a* larvae and controls at 4.5 dpf. Statistical significance between two groups was calculated using unpaired two-tailed Student's *t*-tests assuming equal variance. Scale bars: 200 µm.

Development • Supplementary information

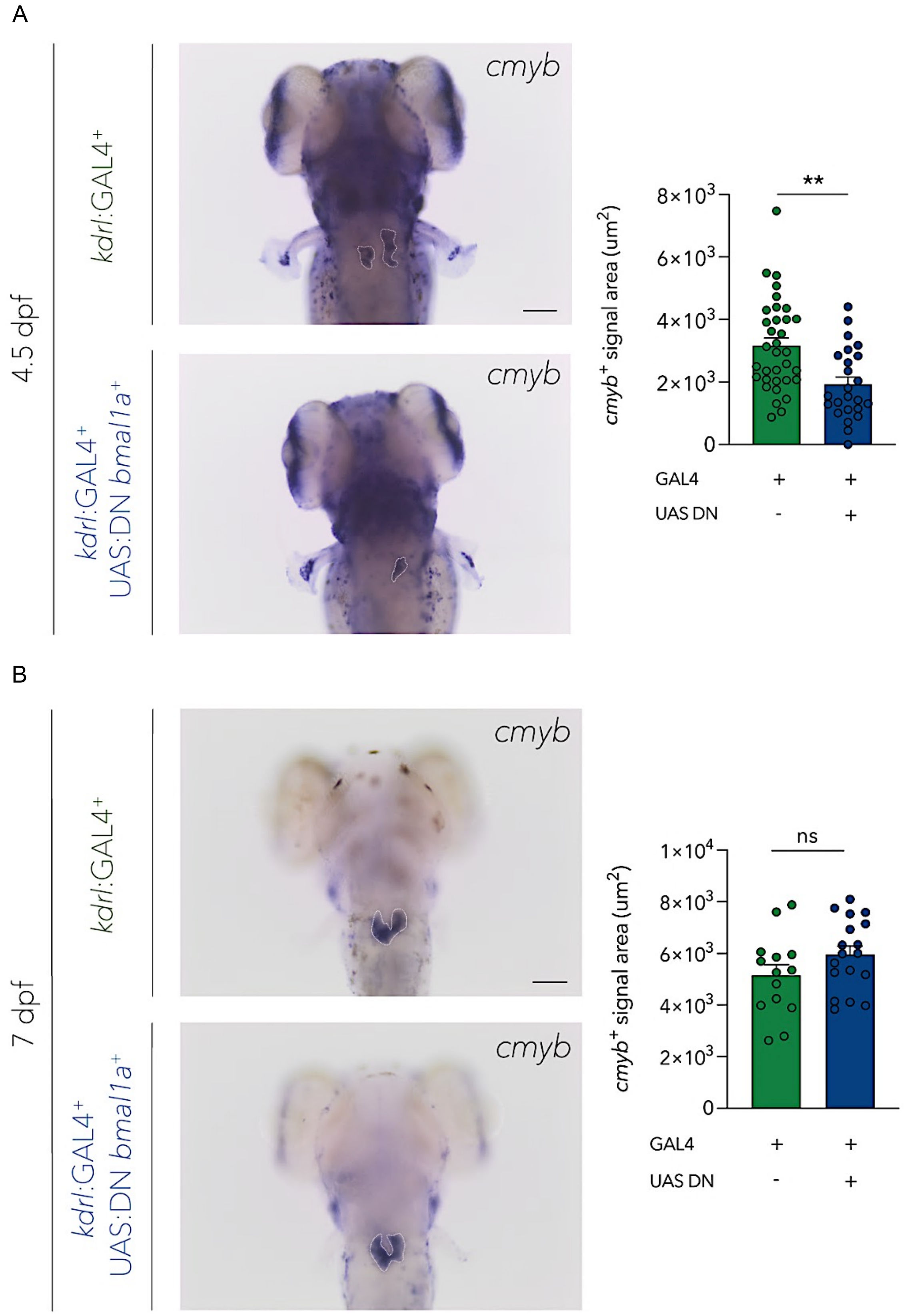

Fig. S8. Endothelial-specific dominant-negative Bmal1a reduces HSPC numbers in the kidney glomeruli at 4.5 dpf but not at 7 dpf. A. *cmyb in situ* hybridisation and quantification in *kdrl:GAL4*;*UAS:DN-bmal1a* embryos and controls at 4.5 dpf. **B.** *cmyb in situ* hybridisation and quantification in *kdrl:GAL4*;*UAS:DN-bmal1a* embryos and controls at 7 dpf. *cmyb*+ cells measured in the kidney glomeruli are marked by white dotted lines in both **A** and **B.** Two independent experiments were carried out for the experiment at 4.5 dpf. One experiment was performed at 7 dpf. Scale bars: 100 μm.

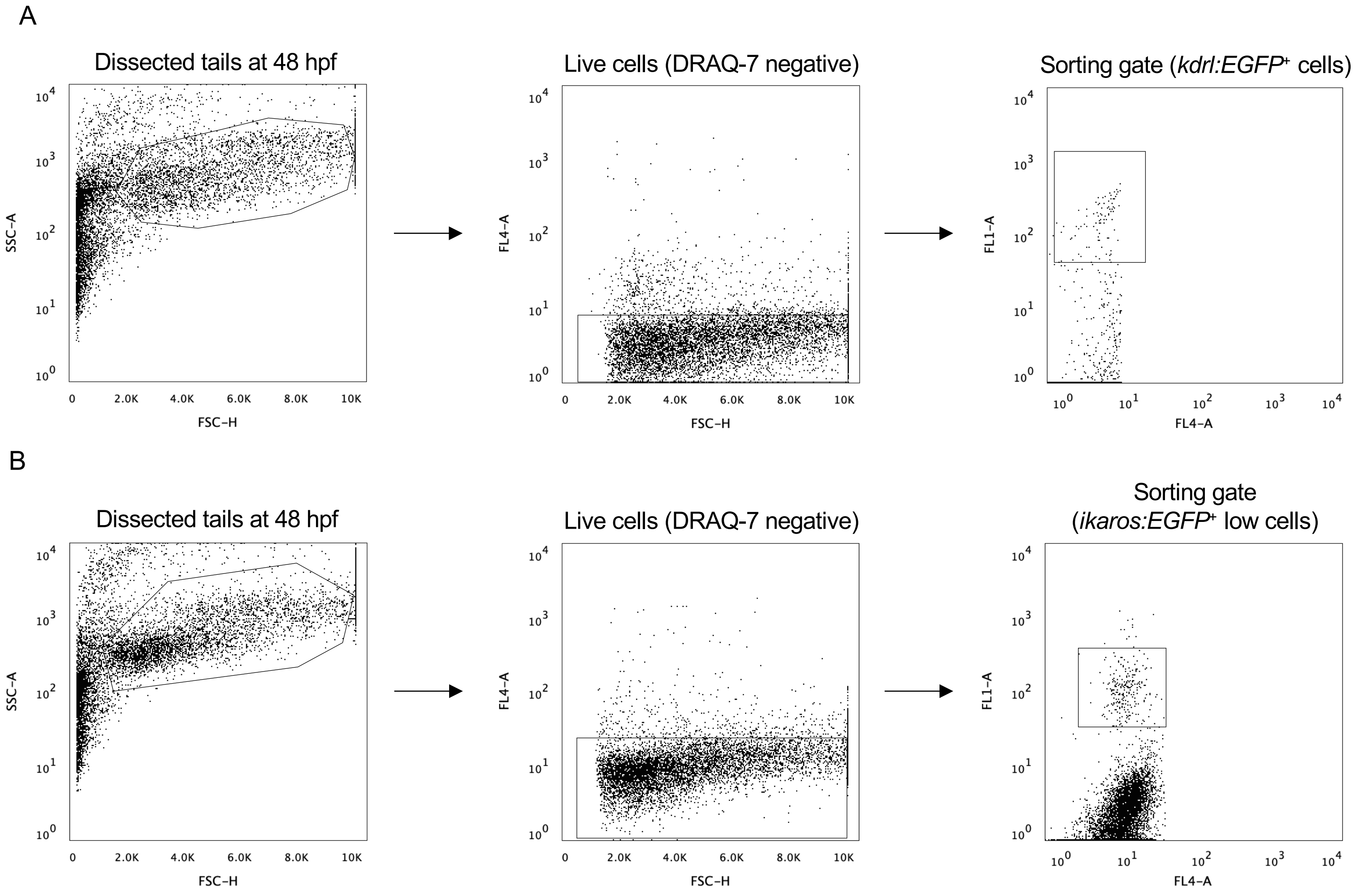

Fig. S9. Fluorescence activated cell sorting (FACS) strategy for sorting endothelial cells and HSPCs from tails of zebrafish embryos at 48 hpf. A. FACS plots to depict the strategy used to sort endothelial cells from the tails of *kdrl:EGFP* embryos at 48 hpf. **B.** FACS plots to depict the strategy used to sort HSPCs from the tails of *ikaros:EGFP* embryos at 48 hpf.

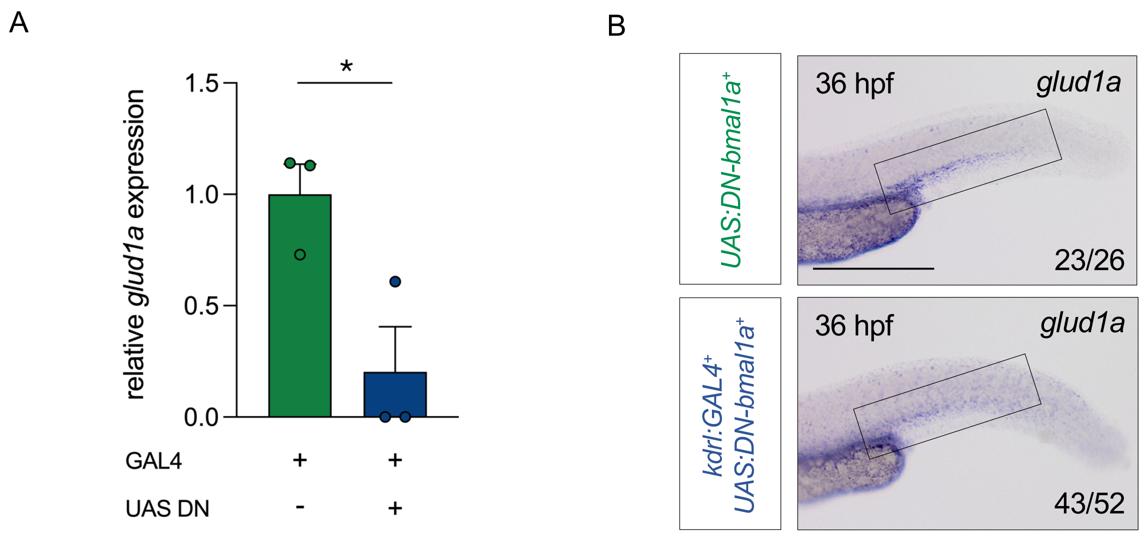

Fig. S10. *glud1a* expression is significantly reduced in endothelial-specific *DN-bmal1a* embryo tail endothelial cells. **A.** qPCR analysis of *glud1a* expression in tail endothelial cells of *kdrl:GAL4;UAS:DN-bmal1a* zebrafish embryos and controls at 36 hpf; *n*=~60 embryos in triplicate for each genotype. **B**. *glud1a in situ* hybridisation in *kdrl:GAL4;UAS:DN-bmal1a* embryos and controls at 36 hpf. Statistical significance between two groups was calculated using unpaired two-tailed Student's *t*-tests assuming equal variance. Scale bar: 200 µm.

Development • Supplementary information

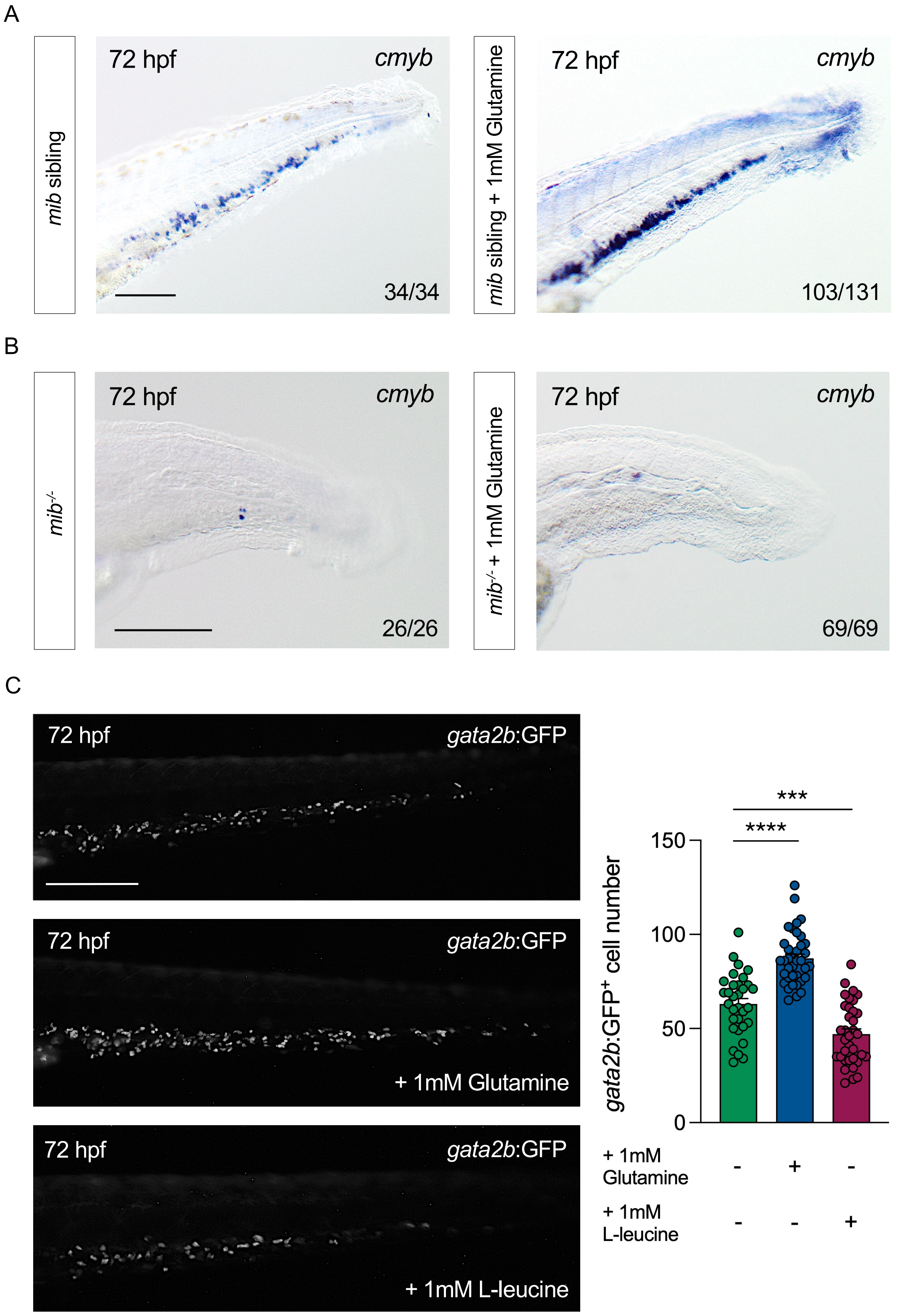

Fig. S11. Definitive HSPC expansion is affected by glutamine and GLUD1A modulation. A. Representative image of *cmyb in situ* hybridisation in *mib* sibling zebrafish embryos at 72 hpf with 1mM glutamine supplementation and controls. **B.** Representative image of *cmyb in situ* hybridisation in *mib*$^{-/-}$ mutant zebrafish embryos at 72 hpf with 1mM glutamine supplementation and controls. **C.** *gata2b*:GFP^{+} cells and quantification in the CHT of *gata2b:KALTA4*$^{+}$*;UAS:lifeact-GFP*$^{+}$ larvae at 72 hpf in controls and when supplemented with 1mM glutamine or 1mM L-leucine. Fractions in **A** and **B** represent the proportion of embryos with WISH signal as in the representative image. Statistical significance between two groups in **C** was calculated using unpaired two-tailed Student's *t*-tests assuming equal variance. Scale bars: 200 μm.

Development • Supplementary information

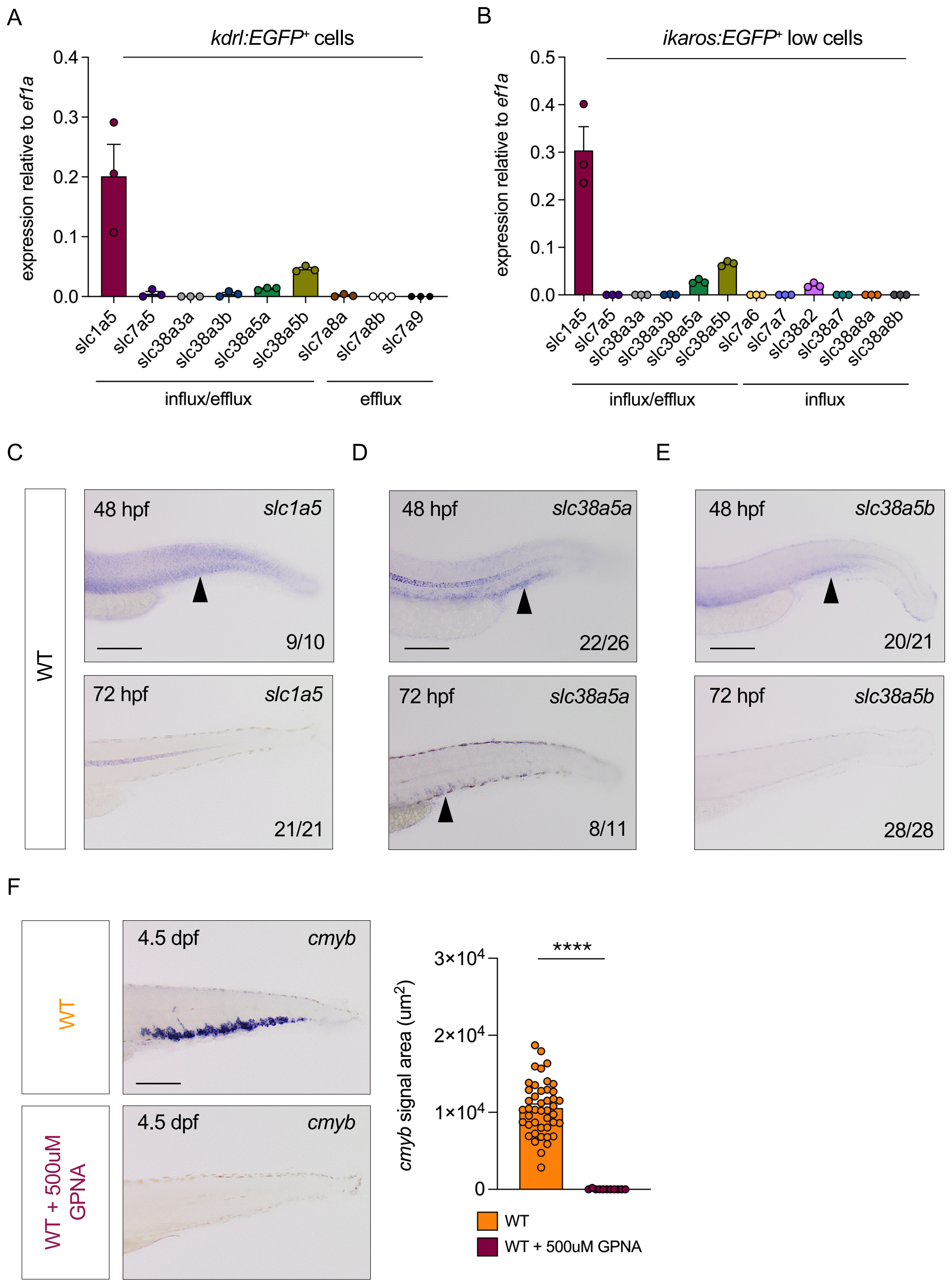

Fig. S12. Glutamine transporter genes are expressed in both endothelial cells and HSPCs. A. qPCR analyses of *slc* gene expression of SLCs able to carry out glutamine efflux in endothelial cells sorted from *kdrl:EGFP* zebrafish embryo tails at 48 hpf; *n*=~80 embryos in triplicate. **B.** qPCR analyses of *slc* gene expression of SLCs genes able to carry out glutamine influx into HSPCs sorted from *ikaros:GFP* tails at 48 hpf; *n*=~80 embryos in triplicate. **C.** *slc1a5 in situ* hybridisation at 48 and 72 hpf in wild-type zebrafish. **D.** *slc38a5a in situ* hybridisation at 48 and 72 hpf in wild-type zebrafish. **E.** *slc38a5b in situ* hybridisation at 48 and 72 hpf in wild-type zebrafish. **F.** *cmyb in situ* hybridisation and quantification in wild-type larvae treated with 500µM GPNA and controls at 4.5 dpf. Statistical significance between two groups in **F** was calculated using unpaired two-tailed Student's *t*-tests assuming equal variance. Scale bars: 200 µm.

Development • Supplementary information

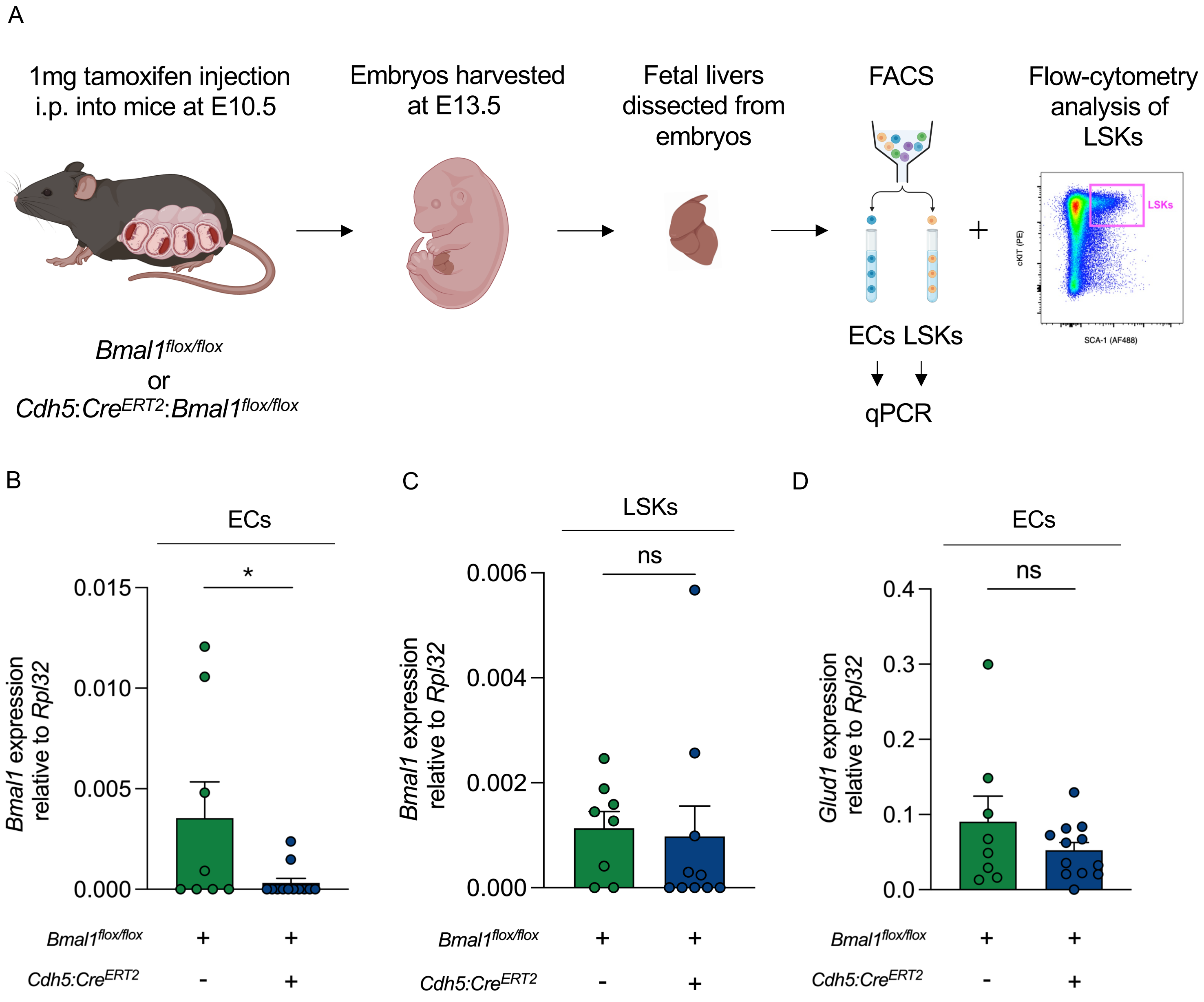

Fig. S13. Endothelial-specific deletion of *Bmal1* in mouse does not result in a difference in HSPC numbers in the fetal liver. A. Schematic of murine experiments. **B.** *Bmal1* expression in fetal liver endothelial cells of *Cdh5*:*Cre*^{ERT2}:*Bmal1*$^{flox/flox}$ embryos and controls at E13.5, as measured by qPCR. **C.** *Bmal1* expression in fetal liver LSKs (HSPCs) of *Cdh5*:*Cre*^{ERT2}:*Bmal1*$^{flox/flox}$ embryos and controls at E13.5, as measured by qPCR. **D.** *Glud1* expression expression in fetal liver endothelial cells of *Cdh5*:*Cre*^{ERT2}:*Bmal1*$^{flox/flox}$ embryos and controls at E13.5, as measured by qPCR. Statistical significance between two groups was calculated using unpaired two-tailed Student's *t*-tests assuming equal variance. Created in BioRender by Petzold, T. (2026) https://BioRender.com/3hkrd9o. This figure was sublicensed under CC-BY 4.0 terms.

Development • Supplementary information

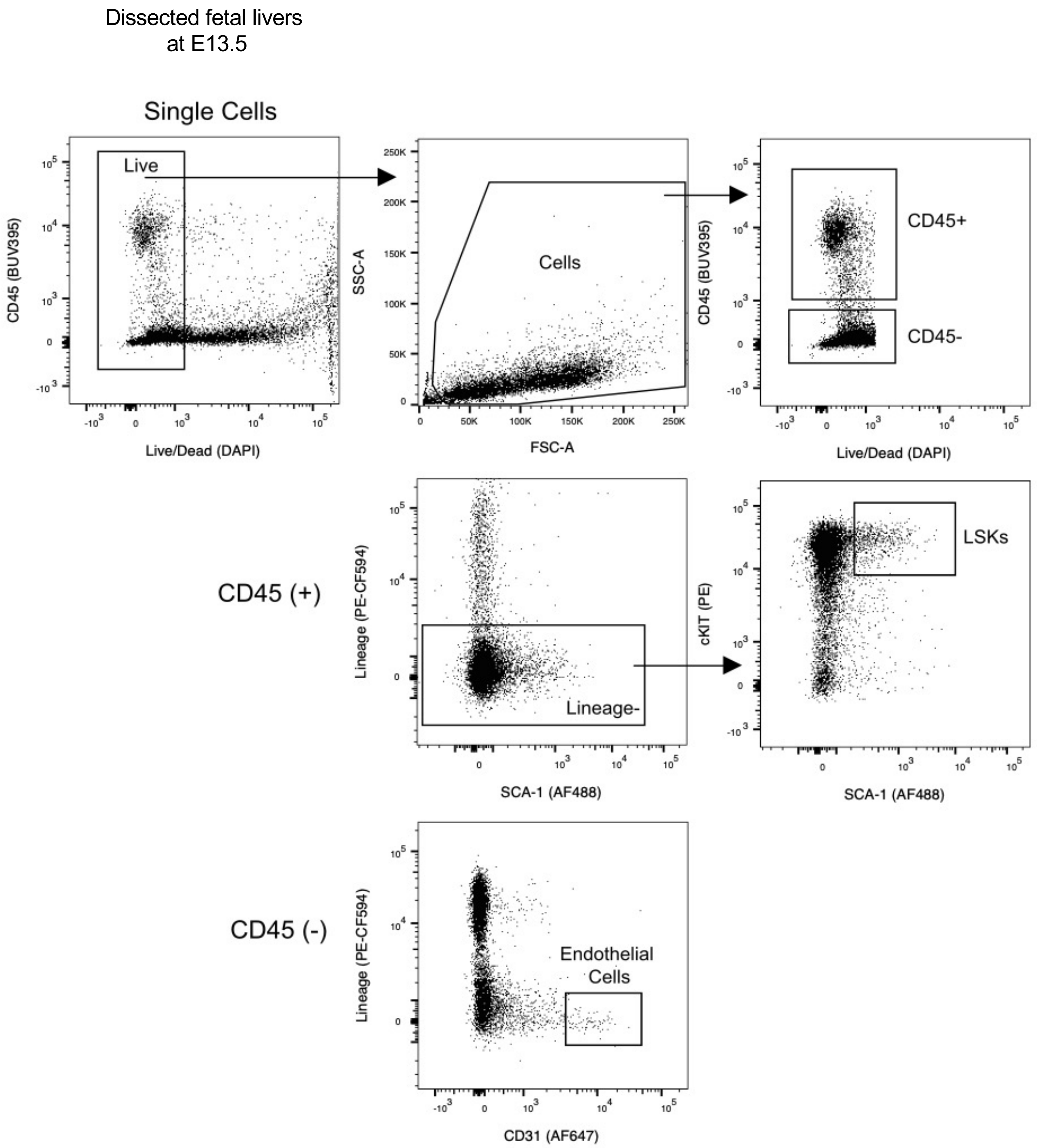

Fig. S14. Fluorescence activated cell sorting (FACS) strategy for sorting LSKs (HSPCs) and endothelial cells from mouse embryo fetal livers at E13.5. FACS plots to depict the strategy used to sort Lin$^-$SCA-1$^+$ cKIT$^+$ LSKs and CD31$^+$ endothelial cells from mouse embryo fetal livers at E13.5.

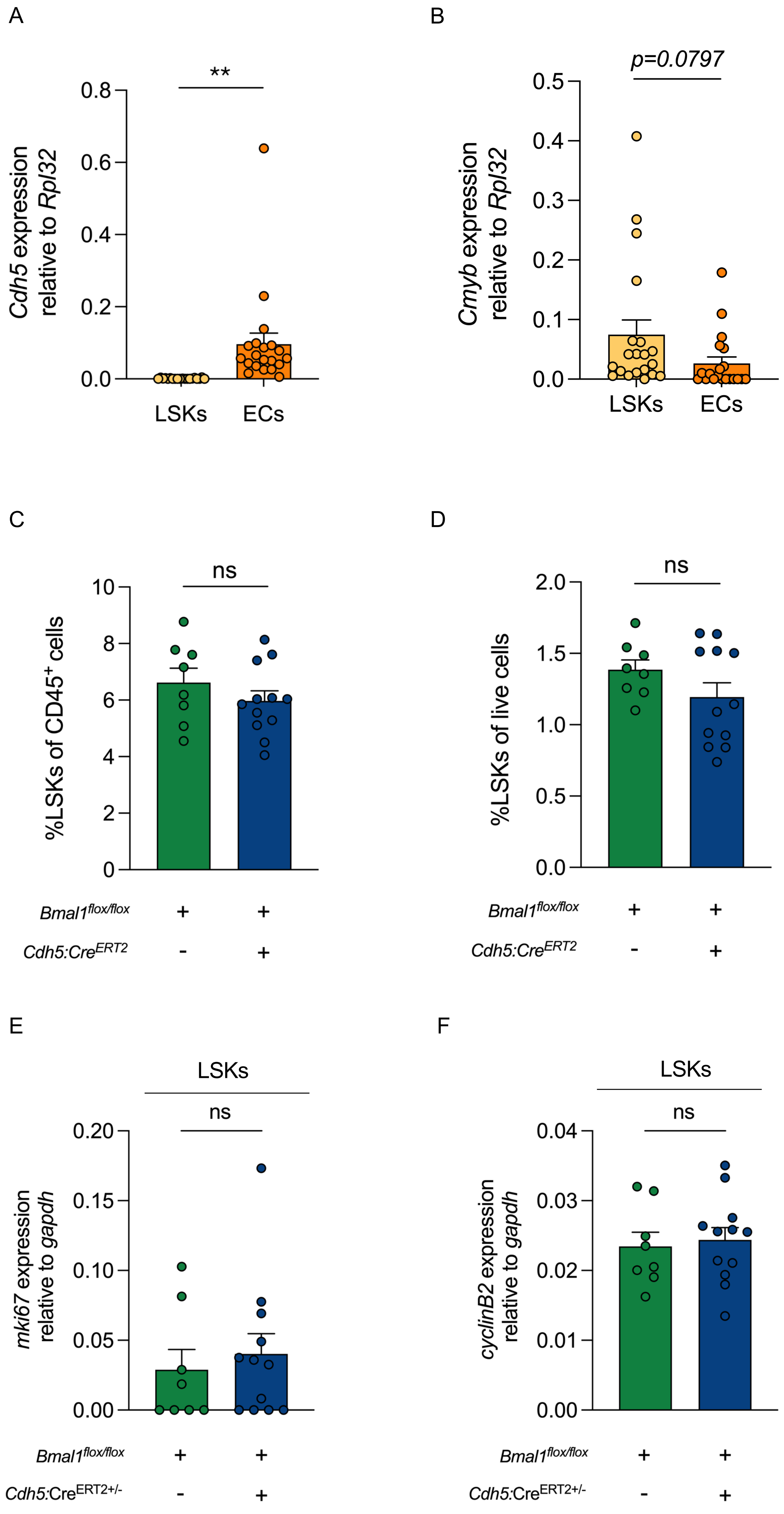

Development • Supplementary information

Fig. S15. Endothelial-specific deletion of *Bmal1* in mouse does not alter LSK (HSPC) numbers in the fetal liver or *Bmal1* expression in LSKs. A. *Cdh5* expression in mouse fetal liver LSKs and endothelial cells at E13.5, as measured by qPCR. **B.** *Cmyb* expression in mouse fetal liver LSKs and ECs at E13.5, as measured by qPCR. **C.** %LSKs of $CD45^{+}$ cells in *Cdh5*:*Cre*^{ERT2}:*Bmal1*$^{flox/flox}$ embryos and controls at E13.5, as measured by flow cytometry. **D.** %LSKs of live cells in *Cdh5*:*Cre*^{ERT2}:*Bmal1*$^{flox/flox}$ embryos and controls at E13.5. **E.** *Mki67* expression in fetal liver LSKs of *Cdh5*:*Cre*^{ERT2}:*Bmal1*$^{flox/flox}$ embryos and controls at E13.5, as measured by qPCR. **F.** *Ccnb2* expression in fetal liver LSKs of *Cdh5*:*Cre*^{ERT2}:*Bmal1*$^{flox/flox}$ embryos and controls at E13.5, as measured by qPCR. Statistical significance between two groups was calculated using unpaired two-tailed Student's *t*-tests assuming equal variance.

Development • Supplementary information

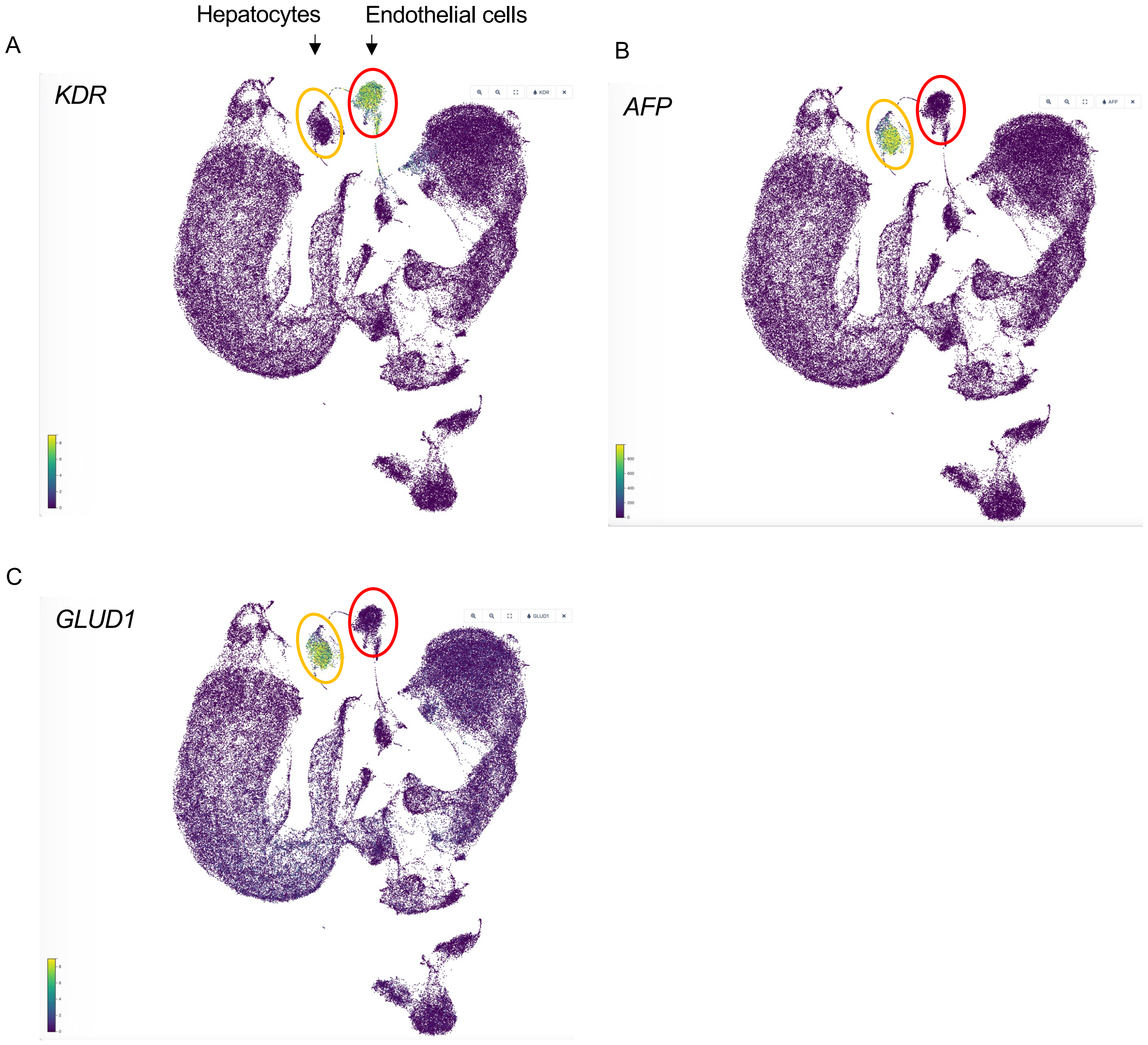

Fig. S16. *GLUD1* is expressed specifically in hepatocytes in human fetal liver. A. *KDR* expression defines the endothelial cell cluster. **B.** *AFP* expression defines the hepatocyte cell cluster. **C.** *GLUD1* is specifically expressed in hepatocytes of the human fetal liver. Yellow and red ovals denote the hepatocyte and the endothelial cell clusters respectively.

Development • Supplementary information

Table S1. Primers used for in situ hybridization probe generation

Gene	Forward	Reverse
bmal1a	AGAACAGCTGTCATCGTCAG	TCAAGCATACTGCCCGAAAC
bmal1b	CTTCATTAGTGCCTACCTGC	ACTCATAGAAAGACGTGCCC
clocka	TCATCTGTACAGACAGCAGG	TGCTGCTGTTGGAGTTGTTG
glud1a	ACAGCCAACCGGTATAACCT	ATCTGTCCCAAGAAGCAGCA
glula	ATCTCCCATTTACTGGACTG	TGCGACTTTGTACCGCACAA
glulb	GCTAGCATACGAATTCCACG	AGTCAGCGCCATACAAGTAC
slc1a5	CTGGACCCAAACCTTGTGAA	CCGGCATCTGAGGTGTTTTA
slc38a5a	TGGGTCAATGGAAAGCAGTC	GGGGGGCTAATAATTCTGAC
slc38a5b	GAGAGAATCGAAGTGGAGGA	TGGGGACAACAGAAAACTGG

Table S2. Primers used for generating dominant-negative bmal1a lines

line	Forward	Reverse
DN-bmal1a	AAGAATTCACCATGGCAGACC AAAGAATGGA	AACTCGAGTTATCCTTCAAG CATACTGCCC
R88A-DN-bmal1a	GGCACACAGTCAAATAGAGAA GAGAGCAAGGGATAAAATGAA CAG	CTGTTCATTTTATCCCTTGCT CTCTTCTCTATTTGACTGTG TGCC

Table S3. Primers used for genotyping

Primer	Forward	Reverse
UAS	AAAGCGGCCGCGGGATCACG CGGCCATCA	AAAACTAGTCGTGTGGAGG AGCTCAAAG
Cre	CGATGCAACGAGTGATGAGG	CGCATAACCAGTGAAACAG C
Bmal1 (flox)	ACTGGAAGTAACTTTATCAAA CTG	CTGACCAACTTGCTAACAAT TA

Table S4. qPCR Primers used in this study

Gene	Forward	Reverse
bmal1a	AAGACATTACGAGGGGCCAC	AGAGGAAACCATCAGCAGCC
bmal1b	ATGGCCGTCCAGCATATGAA	TGGCCAATCAGGTCATTCTG
per2	CCTTCTCGAGACATCCAGAA	ATGCACGCCTTGATGTCGAT
glud1a	GTGCAGTTGTAGATGTGCCT	AACGTCTTGTCTCCAAACCC
slc1a5	GCTGCTTTTCAGTCGTATGC	AGGTGCATACCACATGATCC
slc7a5	ACTCCTCTGCCTTCACTCAT	GGGCAGCAGAATATTGACCT
slc38a3a	CCTCTTCAACCTCAACTCAC	GCTCGGCTTCTACCTTATCA
slc38a3b	GCTGCATGGTTTTCTTCCTC	GATGGTGTAAGCAGTCTGTG
slc38a5a	CATCAACCCACAGACTGCAT	CAAAGATAGCAGTCAGCAGG
slc38a5b	ACGCAATCCCACAAAGAGAC	AACAGAAGCTGAAGAAGGGC
slc7a8a	GAACTGGTCGACCCTTATGT	ACCAAACGTCACAGCTACAG
slc7a8b	CATCCCAGCTTTGCTCATCA	CAAGCTCACTTTGATTGGCC
slc7a9	CACCTTCTCCAGCTCTAATG	ATGACTATCGGCACCTTGAC
slc7a6	GAAAGGAACCTTCCTCTGTC	TCCCACGAAAAACAACCTGG
slc7a7	TATTCCAGGGTTACACCCAG	CAGAGGGAGATTTCTCTCTG
slc38a2	AATATAGCCCTGGATCTGCG	CTCCTTCATTGGCCGTTTTG
slc38a7	CTTCAGATGTGCATGATGGC	TTATTGCGCCGATCAGCTTG
slc38a8a	TTTCAGTGTCATGAGGCCTG	GGATCACAGATCTTCCAAGC
slc38a8b	AGATGGTGAGTCTGGTGTTC	CGTACAGAGACAGACACAAC
glula	CTGCCAGTTCTCAGTTGAGT	GGTGGTTGGTTTCTGCAGTT
glulb	CCTGCAGAAACCAATCTTCG	GAAAGGATGACCATCTGTGC

Development • Supplementary information

Table S4. continued.

Gene	Forward	Reverse
Bmal1	AGAGGTGCCACCAACCCATA	TGAGAATTAGGTGTTTCAGTTC GTCAT
Mki67	GAAGTCAAAGAGCAAGAGGC	CTTTCCAAGGGACTTTCCTG
Ccnb2	TGGCGAAGAAACCTCAGAAC	CTCTCTTCCTTCATGGAGAC
Glud1	AAGGGAGGTATCCGTTACAG	TAAAACCCTTCTTGGCCAGC
Glul	AACCTGCAGAGACCAACTTG	AAGCCATTGGAAGGCCAACC
Cdh5	CCCGTCTTTACTCAATCCAC	TGGGTTTGATGATACCCTCG
Cmyb	AATTATCTGCCCAACCGGAC	GCTTCGGACCATATTTCTGG
Rpl32	ACAATGTCAAGGAGCTGGAG	TTGGGATTGGTGACTCTGATG

Table S5. Differentially expressed genes in FACS-sorted CHT ECs from *kdrl:GAL4;UAS:DN-bmal1a;kdrl:EGFP* embryos and controls at 36 hpf, as determined by RNA-sequencing.

Gene	logFC	logCPM	PValue	FDR
ngs	-4.2227112	6.294271	2.78E-20	2.84E-16
zic2a	-13.462472	4.05282394	3.34E-16	1.71E-12
nr4a1	-2.5461074	8.11258794	7.82E-16	2.67E-12
stab2	-1.9659453	10.1521358	2.62E-12	6.69E-09
hbbe1.1	1.91447154	9.86835633	1.28E-11	2.62E-08
aplnrb	-1.8823056	8.45007857	3.38E-10	5.76E-07
sik1	-1.8327877	7.88526296	6.36E-09	9.29E-06
si:ch211-216l23.1	-2.2823842	6.48303109	1.18E-08	1.51E-05
ptgdsb	2.17830784	6.62134324	1.39E-08	1.58E-05
smyhc1	4.33630728	4.59961487	6.62E-08	6.78E-05
cxcl12a	-2.5511105	5.89194563	9.97E-08	9.27E-05
wu:fc13c02	-9.7672145	3.31158611	1.92E-07	0.00016332
frem1b	-1.6236739	7.87019329	2.35E-07	0.000185
dusp6	-1.7808807	7.08233982	2.66E-07	0.0001946
ctssa	-10.085346	3.20651378	2.88E-07	0.00019631
LOC795545	-1.3814214	9.93810679	6.61E-07	0.00042242
tle2	-1.8720053	6.60458259	9.31E-07	0.00056006
nr2f2	-1.9603837	6.34853658	1.69E-06	0.00091012
egr1	-2.0423472	6.18489191	1.69E-06	0.00091012
capn2b	-10.459604	2.95071324	1.89E-06	0.00096503
hbbe3	1.35327496	9.10362903	2.20E-06	0.00107276
stab1	-1.3210491	9.62758311	2.34E-06	0.00108907
hbae1	1.28745843	10.1074129	3.05E-06	0.00135584
skib	-2.1780644	5.89299564	3.91E-06	0.00164902
slit3	-1.668227	6.81155323	4.03E-06	0.00164902
si:ch73-173h19.3	-11.991945	2.58945236	5.13E-06	0.00201838
smarcc1b	-8.5224499	3.09332138	8.36E-06	0.00316861
igf2b	-1.4247733	7.58946146	9.34E-06	0.00341326
hapln3	-1.3483609	7.98459912	1.20E-05	0.00422076

Development • Supplementary information

Table S5 continued.

entpd1	-1.5350104	6.92570873	1.40E-05	0.00477225
dct	-11.794301	2.39319136	1.73E-05	0.00569727
sash1a	-1.4526771	7.10920245	2.05E-05	0.00655526
ptrfb	-1.4655101	7.04984696	2.15E-05	0.00666733
nr1d2a	-9.9449156	2.65478555	2.76E-05	0.00831725
flrt3	-1.3230721	7.64312653	3.35E-05	0.00963465
zgc:91999	-7.4565272	3.09902905	3.39E-05	0.00963465
col2a1a	-1.5410953	6.61662363	4.25E-05	0.01176571
ghrb	-1.9204009	5.85182775	4.99E-05	0.0130612
tnw	-2.8881499	4.75933569	5.09E-05	0.0130612
si:ch211-5k11.12	1.13522648	9.3816389	5.18E-05	0.0130612
apoeb	-1.1182255	9.89213685	5.23E-05	0.0130612
znf710a	-1.3288282	7.28693991	5.72E-05	0.01393695
sncga	-3.9252708	4.06357129	7.57E-05	0.01801995
ccdc85a	-11.470047	2.07218449	8.32E-05	0.01853683
flt4	-1.1488373	8.57667548	8.33E-05	0.01853683
fosab	-1.2294515	7.82183536	8.33E-05	0.01853683
hbae3	1.0771241	9.87548144	9.71E-05	0.02113708
glud1a	-1.3051016	7.14805821	0.00010911	0.02325963
fbn2b	-1.3149663	7.09098638	0.00011514	0.02404266
si:ch211-69i14.4	-1.8851976	5.73117113	0.00011783	0.0241127
zcchc24	-1.2066872	7.72374425	0.00013075	0.02581496
spry2	-1.8703444	5.73358479	0.00013119	0.02581496
sema3ab	-1.1772064	7.88471207	0.00014631	0.02824682
lin28a	-8.8878815	2.51873033	0.00015801	0.02947478
cyp26b1	-1.8893798	5.65418847	0.00015844	0.02947478
scpp8	1.23554338	7.3266291	0.00016735	0.03037753
ntmt1	-1.4818366	6.44268729	0.00016923	0.03037753
akap12b	-1.0861426	8.73653694	0.00017413	0.03071826
il1b	-2.0077729	5.47585413	0.00018434	0.03130001
tgfbi	-1.0721957	8.90402292	0.00018597	0.03130001

Development • Supplementary information

Table S5 continued.

il17rd	-11.2423	1.84741532	0.00018805	0.03130001
vwf	4.75498715	3.63264619	0.00018966	0.03130001
pdgfra	-11.223959	1.82911133	0.0001967	0.03166759
atp2a1	-1.2892976	6.99990615	0.00019808	0.03166759
ta	-8.8552072	2.40927091	0.00022605	0.03504386
aqp8a.1	-1.0174992	9.82666529	0.00023203	0.03543533
ces2	-11.111798	1.71843824	0.00025704	0.03798151
mxra8a	-11.101019	1.70836313	0.00026338	0.03798151
hsd17b7	-11.085498	1.69338638	0.00027295	0.03798151
cpped1	-11.083675	1.6906642	0.00027453	0.03798151
stm	-1.2355997	7.10261247	0.0002747	0.03798151
cblc	-11.081277	1.6883886	0.00027602	0.03798151
zgc:165518	-9.4820472	2.22821577	0.00028393	0.03798151
socs3b	-1.4709307	6.32326783	0.00028943	0.03798151
col8a2	-1.320044	6.71255685	0.00029253	0.03798151
sall3b	-11.05418	1.66119419	0.00029414	0.03798151
lgals1l1	-11.049469	1.65673299	0.00029726	0.03798151
me3	-1.9549936	5.45013514	0.00030067	0.03798151
cep128	-11.033951	1.64172523	0.00030792	0.03842249
gpm6aa	-11.007728	1.616281	0.00032673	0.03955676
fosl2	-1.0866873	8.09271064	0.00032788	0.03955676
mhc1zda	-8.4993627	2.40207262	0.00032861	0.03955676
zgc:103530	-10.990438	1.5997528	0.00033955	0.04014961
gpd1a	-1.6993266	5.81883835	0.00034413	0.04014961
jak2b	-1.6526955	5.90573966	0.00034531	0.04014961
hic1	-10.976555	1.58520585	0.00035087	0.04033766
ctnnd2b	-10.962689	1.57137887	0.00036209	0.04114331
fynb	-10.95777	1.56688254	0.00036591	0.04114331
lrrn1	-1.1362806	7.5681016	0.0003831	0.0423112
rac3a	-10.933996	1.54303941	0.00038616	0.0423112
ccdc80	-1.2564882	6.85549113	0.0004036	0.04346987
frs2a	-10.881646	1.49211823	0.00043318	0.04616994
tuba8l3	-1.0591881	8.11275729	0.00044618	0.04706509
gch2	2.41132159	4.80728949	0.00045687	0.04770096
myl10	2.14991059	5.07959741	0.00047787	0.0493893

Development • Supplementary information
